# Real-world safety and clinical outcomes of apixaban in patients with cancer and atrial fibrillation

**DOI:** 10.3389/fcvm.2026.1893856

**Published:** 2026-09-11

**Authors:** Lolwa S. Alabdelmuhsin, Metab F. Alwethairi, Dimah Alharbi, Ohoud A. Almadani, Hisham A. Badreldin, Lama Alfehaid

**Affiliations:** 1 Pharmaceutical Care Services, Ministry of the National Guard-Health Affairs, Riyadh, Saudi Arabia; 2 King Abdullah International Medical Research Center, Riyadh, Saudi Arabia; 3 College of Pharmacy, King Saud bin Abdulaziz University for Health Sciences, Riyadh, Saudi Arabia; 4 Research Informatics Department, Saudi Food and Drug Authority, Riyadh, Saudi Arabia

**Keywords:** apixaban, atrial fibrillation, bleeding, cancer-associated atrial fibrillation, cardio-oncology, direct oral anticoagulants, malignancy, real-world study

## Abstract

**Background:**

Patients with cancer and atrial fibrillation (AF) are at increased risk of both thromboembolism and bleeding. However, real-world data evaluating apixaban use in this population remain limited, particularly in Middle Eastern populations.

**Methods:**

We conducted a retrospective observational cohort study at a tertiary care center in Saudi Arabia, including adult patients with active cancer or a history of malignancy who received apixaban for non-valvular atrial fibrillation (NVAF) with or without venous thromboembolism (VTE) between August 2016 and October 2020. Thrombotic and bleeding outcomes were assessed within 6 months of apixaban initiation. Baseline demographic and clinical characteristics, cancer-related variables, concomitant therapies, thrombotic events, bleeding outcomes, mortality, and apixaban discontinuation were descriptively analyzed. Bleeding events were classified according to the International Society on Thrombosis and Hemostasis criteria.

**Results:**

The final analysis included 72 patients. The mean age was 71.0 ± 9.7 years, and 46 patients (63.9%) were female. Active cancer was present in 65 patients (90.3%), while solid tumors accounted for 76.9% of malignancies. Thrombotic events occurred in 7 patients (9.7%), including ischemic stroke in 3 patients and deep vein thrombosis in 3 patients. Bleeding events occurred in 26 patients (36.1%), including major bleeding in 7 patients (9.7%). The most commonly reported bleeding sites were the upper airway and gastrointestinal tract. During follow-up, 21 patients (28.8%) died, most commonly from cancer-related causes. Excluding treatment cessation at the time of death, apixaban was clinically discontinued in 19 patients (26.4%). Documented clinical reasons for discontinuation included bleeding complications, breakthrough thrombotic events requiring a change in anticoagulation therapy, thrombocytopenia, high bleeding risk, and extreme obesity (BMI >50 kg/m^2^).

**Conclusion:**

Thrombotic and bleeding events were common in this real-world cohort of patients with cancer and AF receiving apixaban. These findings highlight the complexity of anticoagulation management in this high-risk population and support the need for close clinical monitoring and further prospective studies.

## Introduction

Atrial fibrillation (AF) is the most common sustained cardiac arrhythmia and is associated with increased risk of stroke, systemic embolism, heart failure, and all-cause mortality (1). Patients with cancer are particularly susceptible to AF, with observational studies showing a risk approximately 1.5 times higher than in people without malignancy (2). Moreover, AF in cancer patients is associated with an almost twofold increased risk of stroke, especially in the first year after the diagnosis of some cancers, including gastrointestinal, pancreatic, hepatic, and brain cancers (3). The co-existence of AF and malignancy thus constitutes a clinically challenging state with competing thrombotic and bleeding risks.

Management of AF in patients with cancer generally follows the same principles used in the general population with regard to rhythm and rate control strategies. But the decisions about anticoagulation therapy are far more complex. Current guidelines support the use of direct oral anticoagulants (DOACs) over warfarin in eligible patients with non-valvular atrial fibrillation (NVAF) except for moderate to severe rheumatic mitral stenosis or mechanical heart valves (1). Historically, however, patients with active malignancy have been underrepresented in major randomized clinical trials that evaluated DOAC therapy.

Evidence for the use of DOACs in patients with cancer has been derived from subgroup analyses of landmark AF trials. In the ARISTOTLE trial, apixaban showed comparable efficacy and safety compared to warfarin in patients with AF and a history of cancer (4). Similarly, in the ROCKET AF and ENGAGE AF-TIMI 48 trials, malignancy was associated with an increased risk of bleeding and mortality, but thromboembolic outcomes were generally similar between DOACs and vitamin K antagonists (5, 6). Later meta-analyses also failed to detect a statistically significant difference between DOACs and warfarin in terms of recurrent stroke, major bleeding, or mortality outcomes in patients with AF and cancer (7). More recently, an updated meta-analysis demonstrated that DOACs were associated with lower risks of stroke/systemic embolism, major bleeding, and all-cause mortality compared with warfarin in patients with cancer and atrial fibrillation, further supporting their use in this high-risk population (8).

Despite these advances, anticoagulation management in patients with AF and malignancy remains challenging in routine clinical practice. Patients with cancer are at increased risk of both thromboembolic and bleeding events, while chemotherapy, thrombocytopenia, gastrointestinal complications that may impair oral drug absorption, and clinically significant drug–drug interactions with anticancer therapies can further influence the safety and clinical outcomes of DOACs (1, 9–12). Moreover, commonly used risk stratification tools, including the CHA₂DS₂-VASc and HAS-BLED scores, have not been adequately validated in oncology populations and do not account for cancer-specific factors that influence thrombotic and bleeding risk (12). Real-world studies have also demonstrated higher rates of thrombotic and bleeding complications among patients with cancer receiving DOAC therapy compared with non-cancer populations (11).

There is limited existing real-world data on the use of apixaban in patients with cancer and AF, especially in the Middle Eastern population. Accordingly, this study aimed to evaluate the real-world safety and clinical outcomes of apixaban in patients with malignancy and AF with or without venous thromboembolism.

## Method

### Study design and study population

This retrospective observational cohort study included adult patients who received apixaban for AF and had active cancer or a documented history of malignancy at the Ministry of National Guard Health Affairs hospitals in Saudi Arabia between August 2016 and October 2020.

Patients were identified through the institutional electronic health record (EHR) system. Eligible patients were aged ≥18 years, had documented follow-up, and had active cancer or a history of malignancy identified using the International Statistical Classification of Diseases and Related Health Problems, 10th Revision (ICD−10), Chapter II: Neoplasms (C00–C97 and D00–D09). Basal cell and squamous cell skin cancers were excluded.

Patients were excluded if they had incomplete clinical information, moderate-to-severe mitral stenosis, a mechanical heart valve, an inability to confirm apixaban initiation despite an active prescription, documented non-adherence, an incomplete malignancy history, or had undergone thrombectomy, vena cava filter placement, or thrombolytic therapy. Patients were also excluded if their cancer diagnosis occurred more than two years before study inclusion.

Clinical data were collected using a combination of structured EHR data extraction and detailed manual chart review. Collected variables included demographic characteristics, baseline comorbidities, indication for apixaban therapy, apixaban initiation date and dose, prior anticoagulant use, concomitant medications with potential interactions with apixaban, cancer characteristics, thrombotic and bleeding events, mortality, and apixaban discontinuation. Baseline comorbidities, including hypertension, diabetes mellitus, heart failure, prior stroke, myocardial infarction, peripheral artery disease, and previous bleeding events, were identified from physician documentation, problem lists, and ICD-10 diagnostic codes when available.

Active cancer was defined as a newly diagnosed, recurrent, or metastatic malignancy within six months before study inclusion or receipt of active anticancer therapy at the time of inclusion. Patients without active cancer were classified as having a history of cancer. Bleeding events were classified according to the criteria of the Scientific and Standardization Committee of the International Society on Thrombosis and Hemostasis.

Thrombotic and bleeding outcomes were validated through physician documentation, imaging reports, laboratory findings, discharge summaries, and hospitalization records. Potential drug–drug interactions with apixaban were assessed using prescribing information from the United States Food and Drug Administration (FDA) and the European Medicines Agency (EMA). Because medication adherence could not be directly assessed retrospectively, patients with documented non-adherence or inability to confirm apixaban initiation were excluded from the study.

Patients were followed from the date of apixaban initiation until apixaban discontinuation, death, or the end of the study period, whichever occurred first. Given the exploratory retrospective design, no formal sample size calculation was performed.

The study protocol was approved by the Institutional Review Board of King Abdullah International Medical Research Center (KAIMRC) (IRBC/0131/21) in January 2021. Given the study's retrospective nature and the use of de-identified data, the Institutional Review Board waived the requirement for informed consent.

### Endpoints

The primary thrombotic endpoint was the occurrence of thromboembolic events within six months of apixaban initiation in patients with malignancy and AF with or without venous thromboembolism (VTE). The primary safety endpoint was the occurrence of bleeding events within six months. Secondary outcomes included all-cause mortality and apixaban discontinuation, including the documented reasons for discontinuation.

### Statistical analysis

Statistical analyses were performed using SPSS version 21.0 (IBM Corp., Armonk, NY, USA). Descriptive statistics were used to summarize baseline characteristics and clinical outcomes. Categorical variables were reported as frequencies and percentages, while continuous variables were presented as mean ± standard deviation (SD) or median with interquartile range (IQR), as appropriate.

The proportions of patients experiencing thromboembolic events, bleeding events, major bleeding, all-cause mortality, and apixaban discontinuation were reported with 95% confidence intervals calculated using the Wilson score method. Exploratory univariate analyses were performed to evaluate factors potentially associated with bleeding events and all-cause mortality. Categorical variables were compared using Fisher's exact test because of the small sample size and low event counts. Odds ratios with 95% confidence intervals were calculated where appropriate. Multivariable regression was not performed because of the limited number of outcome events. A two-sided *p*-value < 0.05 was considered statistically significant.

## Results

### Patient characteristics

**Figure 1:**
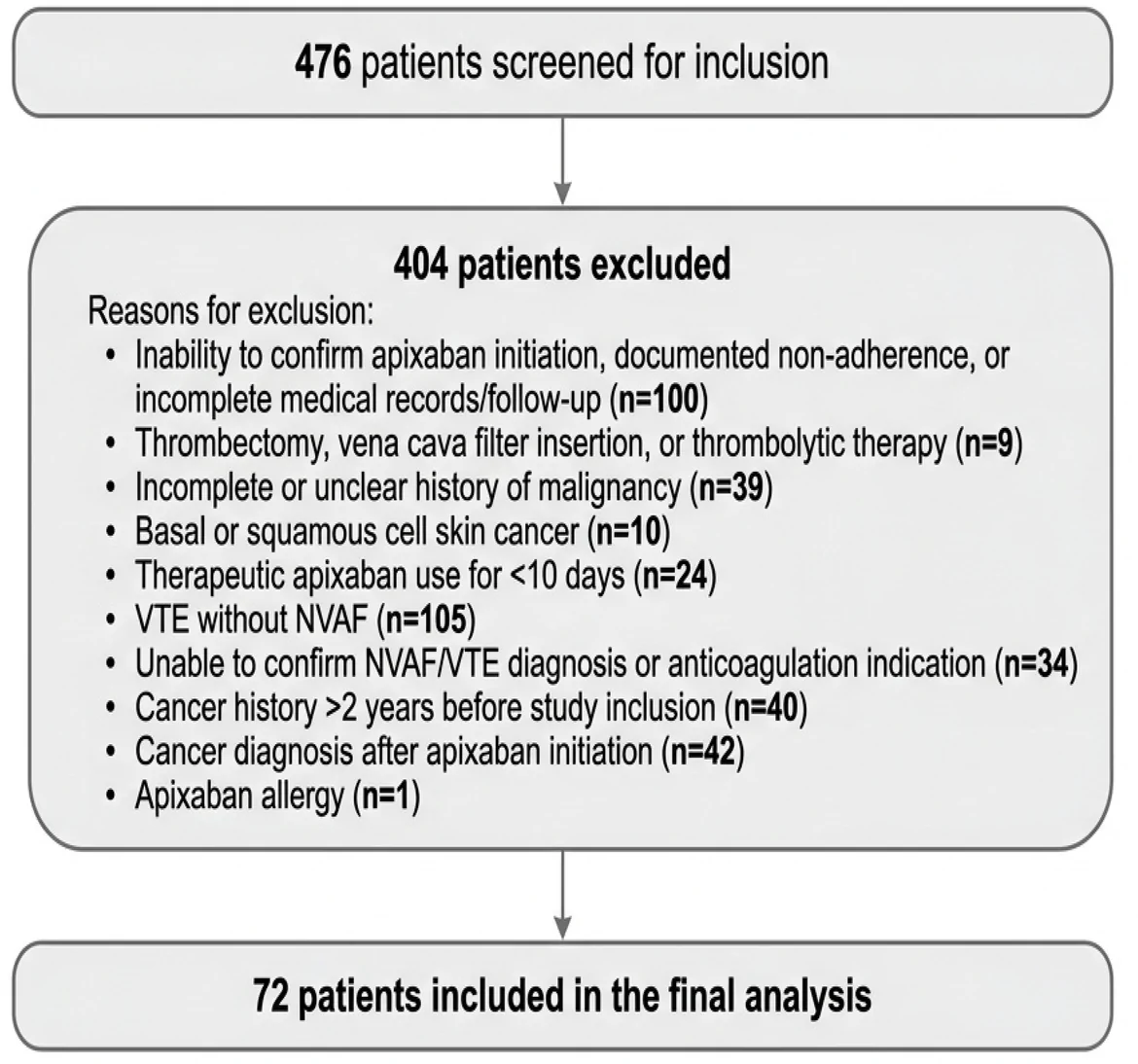
Patient selection flow diagram. NVAF, non-valvular atrial fibrillation; VTE, venous thromboembolism.

A total of 476 patients were screened for eligibility; 72 met the inclusion criteria and were included in the final analysis (Figure 1). Baseline demographic and clinical characteristics are summarized in Tables 1, 2. The mean age of the study population was 71.0 ± 9.7 years, and 46 patients (63.9%) were female. Most patients had preserved renal function, with 59 patients (81.9%) having a baseline creatinine clearance >50 mL/min. Solid tumors represented the predominant malignancy type, accounting for 50 patients (76.9%).

**Table 1:** Baseline demographic and clinical characteristics of patients receiving apixaban (*n* = 72).

Characteristic	Value
Age, years, mean ± SD	71.0 ± 9.7
Female sex, *n* (%)	46 (63.9)
Renal function (creatinine clearance)^a^, *n* (%)	
<30 mL/min	3 (4.2)
30–50 mL/min	10 (13.9)
>50 mL/min	59 (81.9)
Platelet count, *n* (%)	
<100 × 10⁹/L	6 (8.3)
100–349 × 10⁹/L	53 (73.6)
≥350 × 10⁹/L	13 (18.1)
Hemoglobin <10 g/dL, *n* (%)	20 (27.8)
Body mass index, *n* (%)	
<18.5 kg/m^2^	1 (1.4)
18.5–24.9 kg/m^2^	7 (9.7)
25.0–29.9 kg/m^2^	22 (30.6)
≥30 kg/m^2^	42 (58.3)
Appropriate apixaban dose at baseline, *n* (%)	50 (69.4)
ECOG performance status, *n* (%)	
0	2 (2.8)
1	11 (15.3)
2	10 (13.9)
≥3	11 (15.3)
Unknown	31 (43.1)
Active cancer, *n* (%)	65 (90.3)
Recurrent locally advanced or metastatic cancer, *n* (%)	45 (69.2)
Cancer type, *n* (%)	
Solid tumor	50 (76.9)
Hematologic malignancy	15 (23.1)
Indication for anticoagulation, *n* (%)	
NVAF	69 (95.8)
NVAF + DVT	1 (1.4)
NVAF + PE	1 (1.4)
NVAF + DVT + PE	1 (1.4)
CHA_2_DS_2_-VASc score, median (IQR)	4 (2)
CHA_2_DS_2_-VASc score category, *n* (%)	
2	12 (16.7)
3	16 (22.2)
4	22 (30.6)
5	13 (18.1)
6	5 (6.9)
7	2 (2.8)
8	2 (2.8)
Type of atrial fibrillation, *n* (%)	
No data available	47 (65.3)
Paroxysmal	24 (33.3)
Permanent	1 (1.4)
Comorbidities, *n* (%)	
Hypertension	59 (81.9)
Diabetes mellitus	49 (68.1)
Heart failure	23 (31.9)
Prior ischemic stroke, TIA, or MI	9 (12.5)
History of MI, peripheral artery disease, or aortic plaque	6 (8.3)
Prior gastrointestinal bleeding	5 (6.9)
Prior non-gastrointestinal bleeding	8 (11.1)

AF, atrial fibrillation; DVT, deep vein thrombosis; ECOG, Eastern Cooperative Oncology Group; GI, gastrointestinal; IQR, interquartile range; MI, myocardial infarction; NVAF, non-valvular atrial fibrillation; PE, pulmonary embolism; SD, standard deviation; TIA, transient ischemic attack.

a Creatinine clearance calculated using the Cockcroft–Gault equation.

**Table 2:** Cancer characteristics, anticancer therapies, and concomitant medications among patients receiving apixaban (*n* = 72).

Characteristic	Value
Solid tumor type, *n* (%)	
Gynecological	10 (15.4)
Breast	8 (12.3)
Genitourinary	8 (12.3)
Pancreatic or hepatobiliary	7 (10.8)
Lung	5 (6.9)
Upper gastrointestinal	4 (5.6)
Colorectal	3 (4.2)
Other solid tumors	3 (4.2)
Head and neck	2 (2.8)
Hematologic malignancy type, *n* (%)	
Non-Hodgkin lymphoma	7 (9.7)
Multiple myeloma	3 (4.2)
Chronic leukemia	3 (4.2)
Acute leukemia	1 (1.4)
Other hematologic malignancies	1 (1.4)
Anticancer therapies, *n* (%)	
Alkylating agents	21 (29.2)
Monoclonal antibodies	13 (18.1)
Antimetabolites	12 (16.7)
Aromatase inhibitors	9 (12.5)
Kinase inhibitors	9 (12.5)
Taxanes	8 (11.1)
Hormonal therapy	5 (6.9)
Anthracyclines	5 (6.9)
Anti-androgens	4 (5.6)
Topoisomerase inhibitors	4 (5.6)
Vinca alkaloids	4 (5.6)
Immunomodulating agents	3 (4.2)
Proteasome inhibitors	3 (4.2)
Cyclin-dependent kinase inhibitors	1 (1.4)
Venetoclax	1 (1.4)
Other cancer-related therapies, *n* (%)	
Radiation therapy	19 (26.4)
Combination therapy	15 (20.8)
Prior anticoagulation therapy, *n* (%)	31 (43.1)
Concomitant medications with potential interaction with apixaban, *n* (%)	
NSAIDs	15 (20.8)
Clopidogrel	4 (5.6)
Carbamazepine	1 (1.4)
Phenytoin	1 (1.4)
Rifampin	1 (1.4)
Clarithromycin	1 (1.4)

NSAIDs, nonsteroidal anti-inflammatory drugs.

The majority of patients received apixaban for NVAF without concomitant VTE (*n* = 69, 95.8%). Hypertension (81.9%) and diabetes mellitus (68.1%) were the most common comorbid conditions. Active cancer was present in 65 patients (90.3%), while recurrent locally advanced or metastatic disease was identified in 45 patients (69.2%).

### Thrombotic events

Thrombotic events occurred in 7 patients (9.7%) during follow-up (Table 3). Ischemic stroke and deep vein thrombosis (DVT) were the most frequently observed thrombotic complications, each occurring in 3 patients (42.9%), while transient ischemic attack occurred in 1 patient (14.3%). Most thrombotic events occurred early, within the first 3 months of apixaban initiation, with 5 patients (71.4%) experiencing events during this period. When stratified by cancer activity, thromboembolic events occurred in 6 patients (9.2%) with active cancer and in no patients with a history of cancer. Bleeding events occurred in 20 patients (30.8%) with active cancer and 3 patients (42.9%) with a history of cancer. These findings are summarized in Supplementary Table S1.

**Table 3:** Clinical outcomes of apixaban therapy in patients with cancer and atrial fibrillation (*n* = 72).

Outcome	Value	95% CI
Primary effectiveness outcomes		
Total thromboembolic events, *n* (%)	7 (9.7)	4.8–18.7
Type of thromboembolic events^a^, *n* (%)		
Ischemic stroke	3 (42.9)	—
Transient ischemic attack (TIA)	1 (14.3)	—
Proximal deep vein thrombosis (DVT)	2 (28.6)	—
Proximal and distal DVT	1 (14.3)	—
Time to thrombotic event after apixaban initiation^a^, *n* (%)		
0–3 months	5 (71.4)	—
>6 months	2 (28.6)	—
Primary safety outcomes		
Total bleeding events, *n* (%)	26 (36.1)	26.0–47.6
Type of bleeding events^b^, *n* (%)		
Major bleeding	7 (26.9)	4.8–18.7
Clinically relevant non-major bleeding	15 (57.7)	—
Clinically non-relevant non-major bleeding	4 (15.4)	—
Bleeding sites^b^, *n* (%)		
Upper airway	8 (30.8)	—
Hematuria	4 (15.4)	—
Upper gastrointestinal	4 (15.4)	—
Lower gastrointestinal	3 (11.5)	—
Fatal bleeding	2 (7.7)	—
Cutaneous	2 (7.7)	—
Vaginal bleeding	2 (7.7)	—
Abdominal	1 (3.8)	—
Intramuscular	1 (3.8)	—
Mortality outcomes		
All-cause mortality, *n* (%)	21 (29.2)	19.9–40.5
Cause of death^c^, *n* (%)		
Cancer-related	8 (38.1)	—
Other causes	8 (38.1)	—
VTE-related	1 (4.8)	—
Bleeding-related	1 (4.8)	—
Cardiovascular-related	1 (4.8)	—
Unexplained death (PE could not be excluded)	1 (4.8)	—
Unknown	1 (4.8)	—
Clinical apixaban discontinuation		
Apixaban discontinuation, *n* (%)	19 (26.4)	17.6–37.6
Reasons for apixaban discontinuation^d^, *n* (%)		
Thrombosis or bleeding event	6 (31.6)	—
Thrombocytopenia	4 (21.1)	—
Unknown reason	4 (21.1)	—
Suspected thrombosis or bleeding event	3 (15.8)	—
High bleeding risk	1 (5.3)	—
BMI > 50 kg/m^2^	1 (5.3)	—

BMI, body mass index; DVT, deep vein thrombosis; GI, gastrointestinal; PE, pulmonary embolism; TIA, transient ischemic attack; VTE, venous thromboembolism.

a Percentages calculated using patients with thromboembolic events as the denominator (*n* = 7).

b Percentages calculated using patients with bleeding events as the denominator (*n* = 26). Some patients experienced bleeding at multiple sites.

c Percentages calculated using deceased patients as the denominator (*n* = 21).

d Percentages calculated using patients with clinical apixaban discontinuation (excluding discontinuation due to death) as the denominator (*n* = 19).

95% confidence intervals were calculated using the Wilson score method.

### Bleeding events

Bleeding events occurred in 26 patients (36.1%) (Table 3). Among patients who experienced bleeding events, major bleeding occurred in 7 patients (26.9%), clinically relevant non-major bleeding in 15 patients (57.7%), and clinically non-relevant non-major bleeding in 4 patients (15.4%). The most commonly reported bleeding sites were the upper airway, followed by the gastrointestinal tract.

### Mortality and apixaban discontinuation

During follow-up, 21 patients (28.8%) died, with cancer-related mortality representing the most frequently documented cause of death. Excluding treatment cessation at the time of death, apixaban was clinically discontinued in 19 patients (26.4%). Documented clinical reasons for discontinuation included bleeding complications, breakthrough thrombotic events requiring a change in anticoagulation therapy, thrombocytopenia, high bleeding risk, extreme obesity (BMI >50 kg/m^2^), and undocumented reasons.

### Exploratory analysis of factors associated with bleeding events

Exploratory analyses were performed to evaluate factors potentially associated with bleeding events in patients treated with apixaban (Table 4). Gastrointestinal or genitourinary malignancies were associated with significantly higher odds of bleeding events compared with other cancer types (OR 3.09, 95% CI 1.08–8.86; *p* = 0.041). Similarly, baseline hemoglobin <10 g/dL was associated with increased bleeding risk (OR 3.01, 95% CI 1.04–8.68; *p* = 0.048).

**Table 4:** Exploratory factors associated with bleeding events in patients receiving apixaban (*n* = 72).

Variable	Bleeding (*n* = 26)	No bleeding (*n* = 46)	Odds ratio (95% CI)	*p*-value
Gastrointestinal/genitourinary malignancy^a^, *n* (%)	12 (46.2)	10 (21.7)	3.09 (1.08–8.86)	0.041
Hemoglobin <10 g/dL, *n* (%)	11 (42.3)	9 (19.6)	3.01 (1.04–8.68)	0.048
Platelet count <100 × 10⁹/L, *n* (%)	4 (15.4)	2 (4.3)	4.00 (0.67–23.83)	0.187
NSAID use, *n* (%)	8 (30.8)	7 (15.2)	2.47 (0.78–7.83)	0.134
Active cancer, *n* (%)	25 (96.2)	40 (87.0)	3.75 (0.41–34.07)	0.245
Recurrent locally advanced/metastatic disease, *n* (%)	18 (69.2)	27 (58.7)	1.58 (0.57–4.42)	0.382
Inappropriate apixaban dose at baseline^c^, *n* (%)	10 (38.5)	12 (26.1)	1.77 (0.62–5.10)	0.286
Creatinine clearance <50 mL/min^b^, *n* (%)	6 (23.1)	7 (15.2)	1.68 (0.50–5.66)	0.416
Concomitant clopidogrel use, *n* (%)	3 (11.5)	1 (2.2)	5.84 (0.57–59.92)	0.142

Exploratory analyses were performed using Fisher's exact test

Odds ratios and 95% confidence intervals were calculated using univariate analysis.

a Gastrointestinal/genitourinary malignancies included colorectal, upper gastrointestinal, pancreatic/hepatobiliary, and genitourinary cancers.

b Renal impairment was defined as creatinine clearance <50 mL/min.

c Inappropriate apixaban dosing was defined according to FDA-approved dose adjustment criteria.

Percentages were calculated within each bleeding subgroup.

No statistically significant associations were observed between bleeding events and thrombocytopenia, NSAID exposure, renal impairment, active cancer status, metastatic disease, inappropriate apixaban dosing, or concomitant clopidogrel use. However, several of these variables demonstrated numerical trends toward increased bleeding risk.

### Exploratory analysis of factors associated with mortality

Exploratory analyses were performed to identify factors potentially associated with all-cause mortality during follow-up (Supplementary Table S2). Patients with an ECOG performance status ≥3 demonstrated a numerically higher mortality rate than those with lower ECOG scores (54.5% vs. 24.2%; OR 3.68, 95% CI 0.98–13.81; *p* = 0.069). Similarly, concomitant clopidogrel use was associated with a numerically higher mortality rate (75.0% vs. 26.1%; OR 8.33, 95% CI 0.81–85.35; *p* = 0.072), although this association did not reach statistical significance. No statistically significant associations were observed between all-cause mortality and active cancer status, recurrent locally advanced or metastatic disease, renal impairment, baseline anemia, thrombocytopenia, gastrointestinal/genitourinary malignancies, inappropriate apixaban dosing, or the occurrence of thrombotic or bleeding events during follow-up. Given the relatively small sample size and limited number of outcome events, these findings should be considered exploratory and hypothesis-generating.

## Discussion

In this retrospective real-world cohort of malignancy patients with AF treated with apixaban, thrombotic and bleeding events were observed frequently during follow-up. Overall, the rates of thrombosis, major bleeding, clinically relevant non-major bleeding, mortality, and apixaban discontinuation appeared numerically higher than those reported in landmark randomized trials and previously published observational studies assessing DOACs in patients with AF and cancer (4–7, 13, 14).

In our cohort, the incidence of thrombotic events was 9.7%, and most occurred within the first 3 months of apixaban therapy. The observed thrombotic rate was higher than that reported in previous observational studies assessing patients with active cancer and AF treated with DOAC therapy (11). These findings can be explained by several factors. Most patients in our study had active malignancy, and approximately two-thirds had recurrent locally advanced or metastatic disease, both of which are associated with increased cancer-related hypercoagulability. Moreover, cancer-related inflammation, immobility, endothelial dysfunction, and exposure to anticancer therapies may further increase the thrombotic risk despite anticoagulation therapy.

Bleeding complications were also common in this cohort. Overall, 26 patients (36.1%) experienced bleeding events. Among patients with bleeding, 26.9% had major bleeding, 57.7% had clinically relevant non-major bleeding, and 15.4% had clinically non-relevant non-major bleeding, corresponding to overall incidences of 9.7%, 20.8%, and 5.6%, respectively, in the total study population. These bleeding rates were higher than those reported in the ARISTOTLE trial and other retrospective studies evaluating anticoagulation in patients with AF and malignancy (4, 13). Importantly, randomized controlled trials of DOACs have generally enrolled selected patient populations with fewer comorbidities and lower rates of advanced malignancy than those seen in routine clinical practice. In contrast, most of the patients in our cohort had active cancer, several comorbid conditions, and exposure to systemic anticancer therapies, which may increase the susceptibility to bleeding.

We observed a predominance of upper airway and gastrointestinal bleeding, which may reflect the complex interplay between cancer location, mucosal vulnerability, thrombocytopenia, and drug-drug interactions with systemic anticancer therapies. Furthermore, many patients received concomitant therapies that could have pharmacokinetic interactions with apixaban, such as nonsteroidal anti-inflammatory drugs and CYP3A4/P-glycoprotein modifying agents. Gastrointestinal complications such as nausea, vomiting, and mucosal injury, which are common in cancer patients, may also affect the absorption and tolerability of oral anticoagulants. Other studies evaluating DOAC therapy in oncology populations have also demonstrated an increased risk of bleeding in patients with gastrointestinal and genitourinary malignancies, especially at the site of the primary tumor (15). These findings likely reflect local tumor invasion, mucosal fragility, and tumor-related tissue breakdown. Importantly, observational studies and meta-analyses to date have generally shown that apixaban is associated with a similar or lower risk of gastrointestinal bleeding compared with warfarin and several other DOACs, suggesting that the increased bleeding rates observed in our cohort likely reflect the high-risk characteristics of the included oncology population rather than apixaban therapy alone (11, 13, 16–19).

Additionally, the mortality and apixaban discontinuation rates were relatively high in this cohort. The most common cause of death was related to cancer, probably reflecting the advanced oncologic burden of the population included, rather than anticoagulation therapy alone. The rate of clinical apixaban discontinuation probably reflects the clinically unstable and dynamic nature of oncology patients, in whom bleeding events, breakthrough thrombotic events requiring a change in anticoagulation therapy, thrombocytopenia, disease progression, and changes in goals of care frequently require interruption or modification of anticoagulation therapy. The high rates of bleeding and anticoagulation discontinuation in this cohort are likely related to the complex competing risks that exist in real-world oncology patients who are typically underrepresented in randomized clinical trials.

The exploratory analyses identified several clinically relevant factors that may be associated with bleeding risk in patients with cancer and AF receiving apixaban in the present study. Gastrointestinal and genitourinary malignancies were associated with significantly increased odds of bleeding events. This is biologically plausible given the increased mucosal vulnerability, tumor-related bleeding tendency, and local tissue disruption commonly encountered in these malignancies. Similarly, baseline hemoglobin <10 g/dL was associated with nearly three times the odds of bleeding, suggesting that anemia may be a marker of advanced disease burden, occult bleeding, nutritional deficiency, frailty, or chronic inflammatory status in this population. Baseline anemia has also been identified as a clinically relevant marker associated with increased risk of bleeding during anticoagulation therapy in previous observational studies (20).

In this cohort, thrombocytopenia, NSAID exposure, renal impairment, inappropriate apixaban dosing, and concomitant clopidogrel use were not statistically significant predictors of bleeding, although several variables showed numerical trends for increased bleeding risk. These findings should be interpreted with caution, given the small sample size and low number of outcome events; however, they may be useful to identify clinically vulnerable subgroups who may benefit from closer monitoring during anticoagulation therapy.

Exploratory mortality analyses also showed numerically higher mortality rates in patients with poor functional status (ECOG ≥3) and patients who received concomitant clopidogrel therapy. These associations are not statistically significant but are likely to represent the greater clinical complexity, frailty, comorbidity burden, and advanced oncologic disease in these patients. Together, these findings further underscore the importance of personalized anticoagulation strategies and a multidisciplinary approach to cardio-oncology management of patients with malignancy and AF.

Our findings highlight the challenges of anticoagulation management in patients with AF and malignancy and underscore the importance of individualized risk assessment and close clinical monitoring, particularly during the early period after anticoagulation initiation. These findings are consistent with the recommendations of the International Society on Thrombosis and Haemostasis (ISTH) Scientific and Standardization Committee, which advocate individualized anticoagulation strategies based on thromboembolic risk, bleeding risk, potential drug–drug interactions, and changes in cancer treatment over time (12). Additional caution may be warranted in patients with gastrointestinal or genitourinary malignancies because of their potentially increased risk of bleeding. Given the exploratory nature of this study and its relatively small sample size, these findings should be considered hypothesis-generating and warrant confirmation in larger prospective studies.

The study has several limitations. First, its retrospective single-center design may be subject to selection bias, incomplete documentation, and residual confounding. Second, the small sample size and the low number of outcome events limited our ability to perform multivariable analyses or to identify independent predictors of thrombosis or bleeding. Third, there is no comparator group to compare directly with other anticoagulants or untreated populations. Additionally, patients had variable follow-up lengths, and the results may not be applicable to all oncology populations or to patients on other DOAC agents. Finally, this study specifically evaluated patients with AF with or without VTE treated with apixaban, so the findings should not be extrapolated to patients with cancer-associated thrombosis without AF or to other anticoagulants.

Overall, this study provides real-world data on apixaban use in a high-risk oncology population that is underrepresented in randomized clinical trials. Further, larger prospective multicenter studies are needed to better define the safety and efficacy of apixaban and other DOACs in cancer patients with AF.

## Conclusion

In this retrospective real-world cohort of patients with malignancy and AF taking apixaban, thrombotic and bleeding events were common during follow-up. Bleeding complications were particularly common in patients with gastrointestinal, genitourinary, underlining the importance of careful individual risk assessment and close clinical monitoring in this high-risk group. These findings should be interpreted with caution because of the retrospective design and small sample size. Larger prospective studies are needed to further evaluate the safety and efficacy of apixaban in patients with cancer and atrial fibrillation.

## Data Availability

The raw data supporting the conclusions of this article will be made available by the authors, without undue reservation.
