# Supplementary material for: Real-world safety and clinical outcomes of apixaban in patients with cancer and atrial fibrillation
Source: Front Cardiovasc Med. 2026 Sep 11;13:1893856. doi: 10.3389/fcvm.2026.1893856 (PMC13612253; doi:10.3389/fcvm.2026.1893856)
Supplement: Supplementary file 1 [file Datasheet1.pdf]

Supplementary Table S1. Clinical outcomes according to cancer activity

Outcome	Active cancer (n = 65)	History of cancer (n = 7)
Any thromboembolic event	6 (9.2%)	0 (0.0%)
Any bleeding event	20 (30.8%)	3 (42.9%)
All-cause mortality	19 (29.2%)	2 (28.6%)

Supplementary Table S2. Exploratory factors associated with all-cause mortality in patients receiving apixaban (n = 72)

Variable	Death (n = 21)	Alive (n = 51)	Odds ratio (95% CI)	p-value
Age ≥75 years	8 (38.1)	18 (35.3)	1.13 (0.39–3.23)	1.000
Male sex	7 (33.3)	19 (37.3)	0.84 (0.29–2.46)	0.794
Active cancer	19 (90.5)	46 (90.2)	1.03 (0.18–5.79)	1.000
Recurrent locally advanced/metastatic disease	15 (71.4)	30 (58.8)	1.75 (0.58–5.25)	0.424
Hematologic malignancy	3 (14.3)	13 (25.5)	0.49 (0.12–1.93)	0.365
ECOG performance status ≥3	6 (28.6)	5 (9.8)	3.68 (0.98–13.81)	0.069
Hemoglobin <10 g/dL	9 (42.9)	11 (21.6)	2.73 (0.92–8.13)	0.086
Platelet count <100 ×10⁹/L	1 (4.8)	5 (9.8)	0.46 (0.05–4.19)	0.664
Creatinine clearance <50 mL/min	4 (19.0)	9 (17.6)	1.10 (0.30–4.05)	1.000
Gastrointestinal/genitourinary malignancy	9 (42.9)	16 (31.4)	1.64 (0.58–4.68)	0.418
Concomitant NSAID use	3 (14.3)	12 (23.5)	0.54 (0.14–2.16)	0.528
Concomitant clopidogrel use	3 (14.3)	1 (2.0)	8.33 (0.81–85.35)	0.072
Prior anticoagulant therapy	6 (28.6)	25 (49.0)	0.42 (0.14–1.24)	0.126
Inappropriate apixaban dose at baseline	7 (33.3)	15 (29.4)	1.20 (0.40–3.57)	0.783
Bleeding event during follow-up	9 (42.9)	14 (27.5)	1.98 (0.69–5.73)	0.267
Thrombotic event during follow-up	1 (4.8)	6 (11.8)	0.38 (0.04–3.32)	0.665

Exploratory analyses were performed using Fisher's exact test. Odds ratios and 95% confidence intervals were calculated using univariate analysis. Percentages were calculated within each mortality subgroup.
